# Silicone oil residual after vitrectomy for rhegmatogenous retinal detachment

**DOI:** 10.1038/s41433-022-02210-3

**Published:** 2022-09-20

**Authors:** Hongmei Zhao, Tongjie Cheng, Kaicheng Wu, Jian Yu, Yuan Zong, Qian Chen, Chunhui Jiang, Haohao Zhu, Gezhi Xu

**Affiliations:** 1 Department of Ophthalmology and Visual Science, Eye, Ear, Nose and Throat Hospital, Fudan University, Shanghai, People’s Republic of China; 2 Key Laboratory of Myopia of State Health Ministry, and Key Laboratory of Visual Impairment and Restoration of Shanghai, Shanghai, 200031 People’s Republic of China; 3 NHC Key Laboratory of Myopia (Fudan University), Key Laboratory of Myopia, Chinese Academy of Medical Sciences, Shanghai, 200031 People’s Republic of China; 4 Department of Ophthalmology, Shanghai Fifth People’s Hospital, Fudan University, Shanghai, 200240 People’s Republic of China

**Keywords:** Retinal diseases, Risk factors

## Abstract

**Objective:**

To explore the presence of residual emulsified silicone oil (SO) droplets in patients with rhegmatogenous retinal detachment (RRD) and their possible risk factors.

**Methods:**

Patients who underwent primary pars plana vitrectomy with SO injection for RRD and SO removal at the same eye centre were included. Approximately 10 weeks after SO removal, B-scan ultrasonography was performed, and using ImageJ, the silicone oil index (SOI) was measured, and its possible correlations with other clinical factors were explored.

**Results:**

A total of 101 eyes were included. Residual SO particles were found in all the patients (100%), and the mean SOI was 4.04% ± 5.16% (range 0.06%–19.88%). Multiple linear regression revealed that, among all the clinical factors, axial length (AL) and ocular hypertension (intraocular pressure (IOP) > 21 mmHg or the use of antiglaucoma medications) before SO removal were positively and significantly associated with the SOI (all *P* < 0.05). *P*atients with ocular hypertension after SO removal had a higher SOI, a longer SO duration, a higher IOP before SO removal and a longer AL than those without (all *P* < 0.05).

**Conclusions:**

Patients with a larger AL and higher IOP before SO removal were more prone to have more residual SO droplets, which might in turn lead to an elevated IOP. In these eyes, thorough irrigation or repeated fluid-air exchange might be necessary.

## Introduction

Silicone oil (SO) was first introduced by Cibis et al. [1] in the 1960s and is now widely used in the management of complicated retinal detachment. However, complications such as SO emulsification [2] and glaucoma [3–8] have long been a reported concern. Small residual emulsified SO droplets in the vitreous cavity after SO removal have also been widely reported, especially during ultrasound B-scan exams [9, 10]. Recently, using B-scan images and ImageJ software, Stalmans et al. [11] and Shiihara et al. [12] introduced a method for evaluating residual emulsified SO droplets in the vitreous cavity. These studies encouraged us to explore the presence of residual SO in these eyes, but the number of cases in the primary reports is limited [12]. In this study, a relatively large number of patients were included, and using the methods, the presence of residual emulsified SO droplets in the vitreous cavity was studied, and its possible correlation with other clinical factors was explored. This might help to improve our knowledge of residual SO in these eyes and to identify patients who might need special attention.

## Materials and methods

### Study subjects and ethics statement

This was a single-centre, observational, cross-sectional study. Patients who underwent primary pars plana vitrectomy (PPV) with SO injection for rhegmatogenous retinal detachment (RRD), followed by SO removal at the Eye and ENT Hospital of Fudan University between January 2019 and January 2022, were enrolled in the study.

The study was approved by the Institutional Review Board of the Eye and ENT Hospital of Fudan University and conformed to the tenets of the Declaration of Helsinki. All the patients signed informed consent forms.

Patients who underwent a minimum of 8 weeks of follow-up after SO removal and whose retinas were still attached were included. Patients with a history of diabetes mellitus, previous SO injection, previous intraocular disease (except cataract) before RRD (e.g., glaucoma, uveitis), or elevated intraocular pressure (IOP > 21 mmHg), and who were age < 18 years at the time of primary PPV for RRD were excluded from the study.

### Main ophthalmic measurements

At approximately 10(8–12) weeks after SO removal, each patient underwent a thorough ophthalmic examination, which included assessment of the best-corrected visual acuity (BCVA; logarithm of the minimal angle of resolution[logMAR]), slit-lamp microscopy, dilated fundus examination with a noncontact lens (Maxfield 84 Diopter; Ocular, USA), measurement of IOP by noncontact tonometry, measurement of axial length (AL) using IOL master (version 3.01; Carl Zeiss Meditec, Jena, Germany), and a B-scan exam (detailed below). Their demographic features and clinical histories were also collected, including age, sex, number of PPVs, history of ocular trauma, combined procedures during SO removal, duration of SO in situ, lens status, with/without ocular hypertension before and after SO removal (at the time of B-scan) and others. Ocular hypertension was defined as those with IOP higher than 21 mmHg, or the ones using antiglaucoma medications.

### SO removal surgical procedures

SO (5700 cSt; Bausch & Lomb Inc., Rochester, NY, USA) was used in all cases. During SO removal, after the main bulk of SO was removed by active suction, passive drainage was performed, during which a 23-gauge back flute needle was inserted into the vitreous cavity, and the fluid was drained for 15 min while the infusion pressure was set to 20–25 mmHg (Constellation 5000 Vision System, Alcon Laboratories, Inc., Fort Worth, TX, USA).

### B-scan examination and analysis

B-scan was performed using a 10-MHz B-Scan Probe (AVISO, Quantel Medical, France) with the following settings: Gain = 105 dB, Dyn = 55 dB, Tgc = 10 dB. The patients were asked to look in the nasal direction, and the probe was oriented vertically to the temporal sclera. A clear image containing the largest vitreous cavity and lacking ultrasound reverberations due to poor contact between the probe and the eyeball was taken and saved for further analysis.

**Fig. 1:**
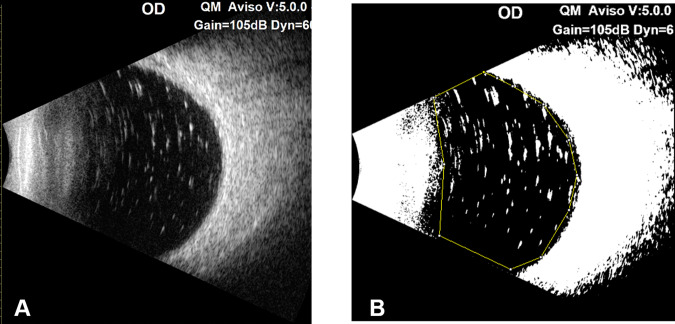
Image processing to quantify the residual silicone oil droplets in the vitreous cavity using B-scan imaging. **A** The ultrasonic B-scan image; (**B**) the image was processed to black and white with a dark background according to the former report, and then a polygon was drawn to represent the vitreous cavity, thus avoiding signals from the retina and nonspecific noises.

Evaluation of the residual silicone oil in situ was performed according to the method described by Stalmans et al. [11] and Shiihara et al. [12]. Briefly, the images were first converted to an 8-bit format. A threshold was set to then convert the image to black and white with a dark background. Then, a polygon was drawn over the vitreous cavity, within which the area of all particles measuring between 25 and 1000 pixels was automatically summed (Fig. 1). The SO index (SOI) was calculated by the following formula:

$${{{\rm{SOI}}}}={{{\rm{area}}}}\,{{{\rm{of}}}}\,{{{\rm{signals}}}}\,{{{\rm{from}}}}\,{{{\rm{hyperechoic}}}}\,{{{\rm{droplets}}}}/{{{\rm{area}}}}\,{{{\rm{of}}}}\,{{{\rm{vitreous}}}}\,{{{\rm{cavity}}}} * 100 \%$$

For the first 20 images, intraobserver repeatability and interobserver reproducibility were evaluated by two observers, who each measured the same scan twice. The intraclass correlation (ICC) coefficient was used to assess repeatability and reproducibility.

### Data and statistical analysis

All analyses were performed using SPSS software version 20.0 (SPSS, Inc., Chicago, IL, USA). Continuous data are expressed as the mean ± standard deviation. ICC coefficients were used to assess repeatability and reproducibility; an ICC coefficient of 0.81–1.00 indicates almost perfect agreement between repeated measurements, and values <0.40 indicate poor to fair agreement.

Spearman’s correlation coefficient, the Mann–Whitney U test and multiple linear regression were used to assess correlations between clinical characteristics and the SOI. The Mann–Whitney U test was also used to assess the differences between patients with/without ocular hypertension after SO removal (at the time of B-scan examination). Statistical significance was defined as a *P* value <0.05.

## Results

A total of 101 eyes (64 right eyes) in 101 patients (62 males) were included in this study. The mean age was 51.29 ± 14.96 years (range 18–86 years), the mean duration of SO in situ was 23.79 ± 12.30 weeks (range 7.00–104.00 weeks), and the mean AL was 26.43 ± 2.76 mm (range 22.02–33.62 mm). At the time of PPV and SO injection, nine patients had choroidal detachment, 15 had a history of ocular trauma, and five had giant retinal tears. For the SO removal procedure, in 69 patients, the procedure was combined with other operations (e.g., phacoemulsification, intraocular lens implantation, or epiretinal membrane peeling). The status of the lens after SO removal was aphakic in 35 eyes, phakic in 29 and pseudophakic in 37.

Residual SO was detected by B-scan in all 101 eyes (100%). The average SOI was 4.04% ± 5.16% (range 0.06–19.88%). The mean ICC coefficients for intraobserver repeatability and interobserver reproducibility were as high as 0.974 and 0.980 (both *P* < 0.001), respectively, which were considered excellent.

Univariate analysis showed that the duration of SO in situ, the AL, the presence of ocular hypertension before SO removal, and the presence of ocular hypertension after SO removal were significantly correlated with the SOI (all *P* < 0.05), while all other parameters were not (all *P* > 0.05) (Table 1). Multiple linear regression revealed that the AL and ocular hypertension before SO removal were positively associated with the SOI (both *P* < 0.05) (Table 2).

**Table 1:** Correlations between the SOI and clinical findings.

Clinical characteristic	SOI	
	r/U/χ^2^ value	*P* value
Age(years)	–0.025	0.803
Male/female	1204.50	0.975
Left eye/right eye	1162.50	0.880
AL (mm)	0.209	0.040^∗^
Duration of SO in situ (weeks)	0.281	0.004^∗^
Numbers of PPV	–0.005	0.962
BCVA before SO injection (logMAR)	0.068	0.512
Time course of SO injection (min)	–0.016	0.905
IOP before SO injection(mmHg)	–0.115	0.252
With choroidal detachment: yes/no	294.00	0.153
With ocular trauma history: yes/no	554.00	0.385
With giant retinal tear: yes/no	127.00	0.077
Ocular hypertension before SOR: yes/no	586.00	0.000^∗^
Lens status before SOR (phakic/aphakic/IOL)	1.780	0.411
BCVA before SOR (logMAR)	–0.131	0.211
Combined other operations during SOR: yes/no	973.00	0.607
Lens status after SOR (phakic/aphakic/IOL)	4.743	0.093
Time course of SOR (min)	0.058	0.760
IOP after SOR (mmHg)	–0.038	0.728
Ocular hypertension after SOR: yes/no	839.00	0.003^∗^

^*^P < 0.05 was considered statistically significant.

Spearman’s correlation coefficients and Mann–Whitney U tests and χ^2^ tests were used to assess correlations between clinical characteristics and SOI.

Ocular hypertension: IOP > 21 mmHg or use of antiglaucoma medications.

*SOI* silicone oil index, *SOR* silicone oil removal, *AL* axial length, *BCVA* best-corrected visual acuity, *logMAR* logarithm of the minimum angle of resolution, *IOP* intraocular pressure.

**Table 2:** Results of multiple linear regression of factors associated with SOI.

Clinical factor	β coefficient (95%CI)	*P* value
Gender: female/male	–0.291 (–2.240, 1.659)	0.768
Age	0.015 (–0.052, 0.082)	0.663
Duration of SO in situ	0.041 (−0.040, 0.121)	0.317
AL	0.453 (0.090, 0.817)	0.015*
Ocular hyperattention before SOR: yes/no	2.856 (0.810, 4.903)	0.007*

*SO* silicone oil, *AL* axial length.

^*^P < 0.05 was considered statistically significant.

Ocular hyperattention means IOP > 21 mmHg or use of antiglaucoma medications.

**Fig. 2:**
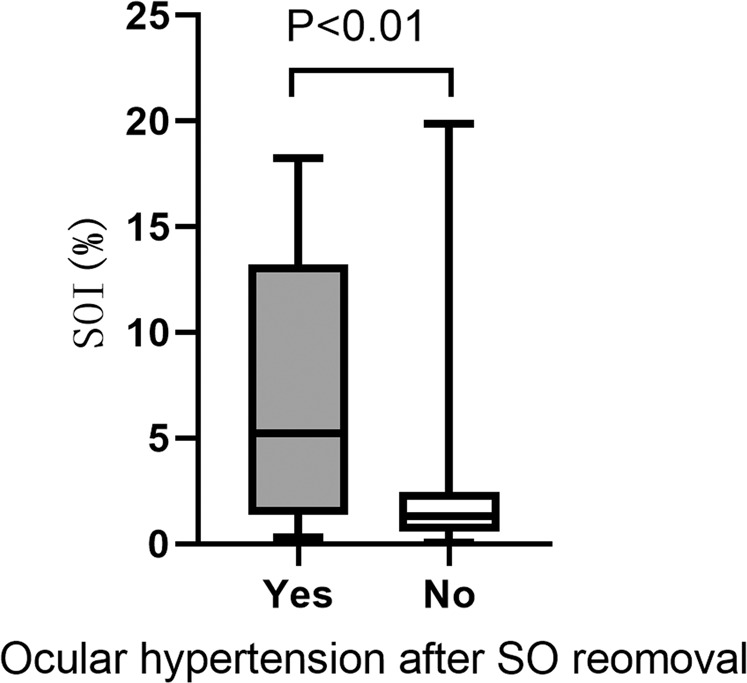
Box and whisker plots comparing SOI between patients with/without ocular hypertension after silicone oil removal. Ocular hypertension: IOP > 21 mmHg or use of antiglaucoma medications.

After SO removal (at the time of the B-scan), 32 patients had ocular hypertension. These patients had a higher SOI, a larger AL, a higher IOP before SO removal and a longer SO duration than patients without ocular hypertension (all *P* < 0.05), and all other parameters were similar between the two groups (all *P* > 0.05) (Table 3 and Fig. 2).

**Table 3:** Findings between patients with/without ocular hypertension after SOR.

Clinical factor	Ocular hypertension		*P* value
	Yes (*n* = 32)	No (*n* = 69)	
SOI	7.15 ± 6.28	2.60 ± 3.82	0.000*
Duration of SO in situ (weeks)	27.72 ± 17.13	21.50 ± 8.85	0.035*
Age (years)	48.75 ± 14.16	52.46 ± 15.28	0.204
AL (mm)	27.82 ± 2.79	25.77 ± 2.50	0.001*
Left eye/right eye	11/21	26/43	0.750
Male/female (n)	13/19	26/43	0.778
Numbers of PPV	1.00 ± 0.00	1.10 ± 0.39	0.120
Time course of SO injection(min)	68.32 ± 24.71	70.39 ± 21.90	0.599
BCVA before PPV + SO injection (logMAR)	2.03 ± 0.95	12.00 ± 0.86	0.901
IOP before SO injection(mmHg)	11.30 ± 3.66	12.51 ± 3.84	0.163
Choroidal detachment, yes/no (n)	4/28	5/64	0.391
Giant retinal tear, yes/no (n)	1/31	4/65	0.567
Ocular trauma, yes/no (n)	3/29	12/57	0.294
Lens status before SOR: non-phakic/phakic (n)	10/22	23/46	0.290
BCVA before SOR (logMAR)	1.33 ± 0.55	1.31 ± 0.56	0.934
IOP before SOR (mmHg)	25.12 ± 6.96	16.54 ± 4.38	0.000*
Combined other operations during SO removal, yes/no (n)	23/9	46/23	0.677
Time course of SO removal(min)	33.22 ± 13.44	34.25 ± 13.37	0.859
Lens status after SO removal: non-phakic/phakic (n)	7/25	22/47	0.364
IOP after SOR (mmHg)	16.35 ± 6.31	15.37 ± 4.59	0.475

*SO* silicone oil, *SOI* silicone oil index, *SOR* silicone oil removal, *AL* axial length, *BCVA* best-corrected visual acuity, *logMAR* logarithm of the minimal angle of resolution.

^*^P < 0.05 was considered statistically significant.

Mann–Whitney U tests and independent-samples *t* tests, χ^2^ tests were used to compare clinical factors between patients with/without ocular hypertension (IOP > 21 mmHg or the use of antiglaucoma medications). Values are expressed as the number of patients or as the mean ± standard deviation.

## Discussion

In this study, using B-scan ultrasonography and ImageJ, residual emulsified SO droplets in the vitreous cavity were studied. A rather large group of patients with RRD was included, one type of SO was used, and B-scan examinations were performed by one ophthalmologist, so the results in this study were more reliable. Residual SO droplets were found in all the patients, and the SOI (the proportional area of SO droplets on B-scan imaging) was positively correlated with AL and ocular hypertension.

Residual emulsified SO droplets in the anterior chamber or the vitreous cavity after SO removal have been observed during slit lamp examinations for many years [13, 10]. Henneken and Machemer [9] first reported highly reflective objects during B-scans. Using ImageJ, Shiihara et al. [12] and Stalmans et al. [11] described a method for quantifying these objects. Compared to other methods, such as the Coulter counter, this method is noninvasive, and could perform a highly cost-effective and highly reproducible in vivo measurement. Above all, this method does not require any specific hardware or software. This provides us with a new way to evaluate residual SO droplets in the clinical setting. However, previous studies only included a limited number of patients with a variety of retinal diseases; as a result, the relationships between residual SO and clinical factors was not fully explored. Therefore, in this study, a relatively large group of patients with RRD were included, and to rule out examiner influence, a B-scan exam was performed by a fixed examiner.

A B-scan exam found emulsified SO droplets in all eyes. This is in accordance with clinical experience and in previous studies [12, 14]. The SOI at this time was 4.04%, which is within the range of previous reports (3.2–7.4%) [12, 14]. This, in another way, suggested the reliability of this method.

In this study, AL, ocular hypertension and SO duration were significantly and positively correlated with the SOI. Recently, Shiihara et al. also reported a correlation between high SOI and AL [12]. The reason for this is not fully understood, but it is possible that eyes with a larger AL have a greater vitreous cavity and intraocular surface area, so a greater volume of SO was injected in these eyes, and the interface between the SO and intraocular fluid was larger. As a result, the surfactants (surface active agents) had a greater opportunity to interact with SO, thus increasing the risk of emulsification. Additionally, in our previous study, we found that the AL was positively and significantly correlated with the total grade of SO emulsification before SO removal [15]. Therefore, more emulsified SO droplets in eyes with greater ALs might be the reason behind this finding. Another factor influencing emulsification is duration [16]. In Federman et al.’s [2] study, the percentages of SO emulsification at 1 month, 2 months, 3 months, 6 months and 1 year were 1%, 6%, 11%, 85% and 100%, respectively. With the same SO removal efficiency, more emulsification means more residual SO, which could explain the positive correlation between the AL, duration of SO tamponade and the SOI found in this study.

The correlation between SO emulsification and elevated IOP has been widely noticed, and many reasons have been proposed. One of them is the migration of SO droplets from the vitreous cavity, which could directly obstruct the trabecular meshwork or cause inflammation [16]. Long-term contact between the emulsified SO droplets and the trabecular meshwork might also result in sclerosis and collapse of the trabecular meshwork. The correlation between SOI and ocular hypertension was also found here. Additionally, eyes with ocular hypertension after SO had a higher SOI than eyes without. These findings, once again, strengthened the important relationship between emulsified SO droplets and a high IOP.

According to the finding this time, special attention must be given to patients with high-risk factors (such as longer AL, ocular hypertension before SO removal), and thorough irrigation or repeated fluid-air exchange should be considered to avoid residual SO droplets, which might in turn lead to high IOP again after SO removal.

Our study was limited by its cross-sectional design, and only one type of SO – 5700 cSt was studied. Additionally, previous studies have revealed that most emulsified droplets are too small in size to be seen on clinical examination [17–19], and resolution of B-scan(especially the 10 MHz used here) is limited, so small droplets could be missed. And, B-scan can only detect SO droplets in the vitreous cavity and cannot detect those that have adhered to the retina or anterior segment. Furthermore, the results of B-scan might be operator dependent, and the SOI only provided the sum-up area of all droplets, the size distribution was not available like other method, for example Coulter counter.

In conclusion, B-scan imaging and ImageJ were used to study residual SO droplets in the vitreous cavity in RRD patients treated with SO tamponade. More droplets were found in patients with greater ALs and those with ocular hypertension before SO removal, and special attention should be given to these individuals during SO removal to ensure more complete removal. Future long term follow-up study should be carried out to see if the SOI changes with time, and to compare the findings of different method - B-scan, Coulter Counter and UBM in the evaluation of SO emulsification. These studies might further improve our knowledge in this field.

## Summary

### What was known before

- Recently, using B-scan images and ImageJ software, researchers introduced a method for evaluating residual emulsified SO droplets in the vitreous cavity.

### What this study adds

- Patients with a larger AL and higher IOP before SO removal were more prone to have more residual SO droplets, which might in turn lead to an elevated IOP.

## Data Availability

The research data used to support the findings of this study are included within the article.
